# Level and course of FEV_1 _in relation to polymorphisms in *NFE2L2 *and *KEAP1 *in the general population

**DOI:** 10.1186/1465-9921-10-73

**Published:** 2009-08-11

**Authors:** Mateusz Siedlinski, Dirkje S Postma, Jolanda MA Boer, Gerrit van der Steege, Jan P Schouten, Henriette A Smit, H Marike Boezen

**Affiliations:** 1 Department of Epidemiology, University Medical Center Groningen, University of Groningen, Groningen, The Netherlands; 2 Department of Pulmonology, University Medical Center Groningen, University of Groningen, Groningen, The Netherlands; 3 National Institute for Public Health and the Environment, Bilthoven, The Netherlands; 4 Department of Medical Genetics, University Medical Center Groningen, University of Groningen, Groningen, The Netherlands

## Abstract

**Background:**

The metabolism of xenobiotics plays an essential role in smoking related lung function loss and development of Chronic Obstructive Pulmonary Disease. Nuclear Factor Erythroid 2-Like 2 (NFE2L2 or NRF2) and its cytosolic repressor Kelch-like ECH-associated protein-1 (KEAP1) regulate transcription of enzymes involved in cellular detoxification processes and *Nfe2l2*-deficient mice develop tobacco-induced emphysema. We assessed the impact of Single Nucleotide Polymorphisms (SNPs) in both genes on the level and longitudinal course of Forced Expiratory Volume in 1 second (FEV_1_) in the general population.

**Methods:**

Five *NFE2L2 *and three *KEAP1 *tagging SNPs were genotyped in the population-based Doetinchem cohort (n = 1,152) and the independent Vlagtwedde-Vlaardingen cohort (n = 1,390). On average 3 FEV_1 _measurements during 3 surveys, respectively 7 FEV_1 _measurements during 8 surveys were present. Linear Mixed Effect models were used to test cross-sectional and longitudinal genetic effects on repeated FEV_1 _measurements.

**Results:**

In the Vlagtwedde-Vlaardingen cohort SNP rs11085735 in *KEAP1 *was associated with a higher FEV_1 _level (p = 0.02 for an additive effect), and SNP rs2364723 in *NFE2L2 *was associated with a lower FEV_1 _level (p = 0.06). The associations were even more significant in the pooled cohort analysis. No significant association of *KEAP1 *or *NFE2L2 *SNPs with FEV_1 _decline was observed.

**Conclusion:**

This is the first genetic study on variations in key antioxidant transcriptional regulators *KEAP1 *and *NFE2L2 *and lung function in a general population. It identified 2 SNPs in *NFE2L2 *and *KEAP1 *which affect the level of FEV_1 _in the general population. It additionally shows that *NFE2L2 *and *KEAP1 *variations are unlikely to play a role in the longitudinal course of FEV_1 _in the general population.

## Background

The mortality and morbidity of Chronic Obstructive Pulmonary Disease (COPD) has been increasing over the past decades and the disease is a fundamental medical and economical problem in Western societies [1]. A genetic predisposition is thought to play a crucial role in the onset of COPD and the heritability of lung function loss that precedes COPD development has been clearly established [2,3]. Several polymorphisms have been identified in association with level of lung function, but subsequent studies have failed to replicate these reported associations [4,5]. So far, only a small subset of polymorphisms has been consistently replicated in their association with COPD development or lung function decline across independent studies or populations [6-11].

Nuclear Factor (Erythroid-derived 2)-Like 2 (NFE2L2 or NRF2) regulates the transcription of numerous antioxidant enzymes in response to oxidant injury, via direct binding to the antioxidant responsive element in the target gene [12-15]. It therefore is a potent candidate gene for excess lung function loss and COPD development.

Kelch-like ECH-associated protein-1 (KEAP1) is a cytosolic repressor of NFE2L2. Oxidative stress causes disruption of the KEAP1-NFE2L2 complex, translocation of NFE2L2 to the nucleus and subsequent induction of the expression of antioxidant genes [16]. It has been shown that *Nfe2l2 *protects mice against elastase-induced [17] and tobacco-induced [18] emphysema. Additionally, the expression pattern of both *KEAP1 *and *NFE2L2 *is different in COPD patients as compared to healthy never- or former- smokers [19,20] and the expression of NFE2L2-regulated antioxidant genes is lower in COPD subjects than in non-diseased controls [21]. Three new polymorphisms have been discovered in the promoter region of *NFE2L2*, but these were not associated with COPD in a Japanese population [22]. One study showed that one of these polymorphisms decreases *NFE2L2 *expression *in vitro *and is associated with development of acute lung injury in a Caucasian population [23]. So far no studies have investigated the role of *NFE2L2 *or *KEAP1 *polymorphisms in relation to the longitudinal course of lung function in the general population.

Therefore, in the current study we investigated whether *NFE2L2 *or *KEAP1 *polymorphisms affect the level and longitudinal course of FEV_1 _(Forced Expiratory Volume in 1 second), both being important risks for COPD [24]. In order to assure consistency of results, we performed this study in two prospective and independent population-based cohorts.

## Methods

### Subjects

Subjects from the Doetinchem cohort study [25], a prospective part of the MORGEN study [26], were included. A sub-sample (n = 1,152 subjects with 3,115 FEV_1 _measurements during 3 surveys: surveys 1993–1997 (n = 1,152), 1998–2002 (n = 1,152), and 2003–2007 (n = 811)), table 1) was randomly selected from the total cohort with spirometry tests and DNA available as described previously [27]. FEV_1 _was measured three times (maneuver performed with a heated pneumotachograph (Jaeger, Germany)) with 5-year intervals according to the European Respiratory Society (ERS) guidelines [28].

**Table 1:** Characteristics of Doetinchem cohort and Vlagtwedde-Vlaardingen cohort

	**Doetinchem cohort** (n = 1,152)	**Vlagtwedde-Vlaardingen cohort** (n = 1,390)
**Total duration of follow-up **(years)	10	25

**Number of visits **(median)	3	7

**The total number of FEV_1 _measurements across all visits**	3,115	8,159

**Follow-up time frame**, years	1997–2007	1965–1990

**Males**, n (%)	541 (47.0)	714 (51.4)

**FEV_1 _change in ml/year**, mean (SD)	-26.2 (33.4)	-20.8 (22.9)

**Last available:**		

**FEV_1 _level in liters**, mean (SD)	3.31 (0.80)	2.86 (0.77)

**Age in years**, median (range)	53.7 (32–76)	52.0 (35–79)

**Never smokers**, n (%)	372 (32.3)	445 (32.0)

**Packyears smoked in ever smokers**, median (range)	13.2 (0.004–84.0)	18.9 (0.1–262.2)

FEV_1 _= Forced Expiratory Volume in 1 second

SD = Standard Deviation

An independent cohort (Vlagtwedde-Vlaardingen; n = 1,390 subjects with 8,159 FEV_1 _measurements during 8 surveys, table 1) was additionally studied. This cohort was prospectively followed for 25 years with FEV_1 _measurements (maneuver performed with a water-sealed spirometer (Lode Instruments, the Netherlands)) every 3 years (following ERS guidelines) [29].

The study protocols were approved by local medical ethics committees and all participants gave their written informed consent.

### Selection/genotyping of Single Nucleotide Polymorphisms (SNPs)

We pairwise tagged *NFE2L2 *and *KEAP1 *with respectively five and three SNPs according to the HapMap CEU genotype data (23a) with an r^2 ^threshold of 0.8 and Minor Allele Frequency (MAF)>5%. We additionally included three novel *NFE2L2 *polymorphisms [22] with MAF>5%: G(-686)A (rs35652124), C(-650)A (rs6721961) and Trinucleotide CCG Repeat (TNR). SNPs were genotyped by K-Bioscience Ltd (UK) using their patent-protected competitive allele specific PCR system (KASPar). The additional file 1 contains details on SNP-selection and *NFE2L2 *TNR genotyping.

### Statistics

#### SNPs in NFE2L2 and KEAP1 and level of FEV_1_

We used Linear Mixed Effect (LME) to study the effects of SNPs and haplotypes (additive genetic model; coded: 0 = homozygote wild type, 1 = heterozygote, 2 = homozygote mutant) on the level of FEV_1 _in both cohorts separately, using all available FEV_1 _measurements across all surveys. This analysis was adjusted for age (defined with natural cubic spline with 4 degrees of freedom in order to take into account varying effects of age on the level of FEV_1 _throughout lifetime), sex, packyears smoked, height and the correlation of FEV_1 _measurements within each subject (random effect assigned to the intercept).

#### SNPs in NFE2L2 and KEAP1 and course of FEV_1_

We studied the effect of SNPs on course of FEV_1 _by introducing the interaction term of SNP × time (defined in relation to the first FEV_1 _measurement and with random effect assigned) into the primary analysis model described above (see additional file 1 for details).

#### Analysis on the pooled cohorts

Finally, we pooled both cohorts, and performed analysis on the level and course of FEV_1 _with additional adjustment for cohort. We studied also two other models (recessive/dominant = mutant/wild type homozygotes compared to the rest genotypes) which were reported in case they showed significant effects in the pooled cohort analysis. Similarly we investigated whether there was a significant interaction between *KEAP1 *and *NFE2L2 *genotypes in relation to the level of FEV_1_, using two-way combinations of genetic effects with the highest statistical power i.e. dominant and additive.

#### Interaction with smoking

Gene by smoking interaction analysis in relation to the level of FEV_1 _was performed on the pooled cohorts using data from single surveys (i.e. second in the Doetinchem cohort and last in the Vlagtwedde-Vlaardingen cohort) in order to ensure the highest cumulative exposure to tobacco smoke and the highest number of subjects analyzed. The following interaction terms in two following regression models were analyzed:

1. SNP by ever/never smoking status in the total population with adjustment for ever-smoking status and genotypes and no adjustment for packyears smoked

2. SNP by packyears smoked within ever smokers with adjustment for packyears smoked and genotypes

P values < 0.05 were considered to be statistically significant (tested 2-sided).

### Software

LME models were run using S-PLUS (version 7.0). Linkage Disequilibrium (LD) plots and Hardy-Weinberg Equilibrium (HWE) tests were performed with Haploview (version 4.1) [30]. We identified, with a probability > 95%, subjects carrying no, one or two copies of a specific haplotype, using the *. out_pairs output file from PHASE software (version 2.1) [31,32]. We used MIX software (version 1.7) [33,34] to meta-analyze results from the Doetinchem, Vlagtwedde-Vlaardingen and British 1958 Birth cohort [35].

## Results

### Genetic structure of *NFE2L2 *and *KEAP1*

There was an excess of *KEAP1 *rs1048290 SNP heterozygotes in the Vlagtwedde-Vlaardingen cohort, which caused a significant deviation (p = 0.01) from HWE (table 2). To eliminate potential genotyping errors as underlying cause of this, we additionally genotyped *KEAP1 *rs9676881 SNP, that is in complete LD with rs1048290 (based on HapMap; distance between the two SNPs = 3.7 kb). This SNP also showed a significant deviation from HWE (p = 0.01; frequency of 50.6% and 12.4% for heterozygotes and homozygote mutants respectively) in the Vlagtwedde-Vlaardingen cohort.

**Table 2:** Characteristics of *NFE2L2 *and *KEAP1 *genotypes in the Doetinchem cohort and Vlagtwedde-Vlaardingen cohort

		**Doetinchem cohort** (n = 1,152)				**Vlagtwedde-Vlaardingen cohort** (n = 1,390)			
**The total SNP call rate**, %		97.5				96.4			

**The total unique haplotype call rate**, %		93.7				90.6			

**Genotypes distribution**, n(%):		Heterozygotes	Homozygotes mutant	MAF	HWE p value	Heterozygotes	Homozygotes mutant	MAF	HWE p value

*NFE2L2*	rs6726395	561 (49.6)	256 (22.7)	47.5	0.91	670 (49.6)	277 (20.5)	45.3	0.82

	rs4243387	210 (18.8)	10 (0.9)	10.3	0.69	191 (14.1)	14 (1.0)	8.1	0.07

	rs1806649	454 (40.5)	72 (6.4)	26.7	0.27	510 (39.1)	83 (6.4)	25.9	0.55

	rs13001694	530 (47.3)	178 (15.9)	39.6	0.74	647 (48.1)	223 (16.6)	40.6	0.82

	rs2364723	499 (44.6)	105 (9.4)	31.7	0.38	574 (42.8)	156 (11.6)	33.0	0.42

	HaplotypeC	326 (30.4)	41 3.8)	18.9	0.72	402 (32.6)	44 (3.6)	19.1	0.42

	HaplotypeD	237 (22.1)	13 1.2)	12.4	0.47	283 (22.9)	14 (1.1)	13.0	0.13

*KEAP1*	rs1048290	507 (45.5)	147 (13.2)	36.0	0.77	671 (50.6)	164 (12.4)	37.6	0.01

	rs11085735	117 (10.4)	5 (0.4)	5.6	0.56	129 (9.6)	2 (0.1)	4.9	0.77

	rs1048287	203 (18.0)	18 (1.6)	10.6	0.14	248 (18.4)	11 (0.8)	10.0	0.51

	HaplotypeA	520 (47.9)	197 (18.1)	57.7	0.64	659 (51.2)	222 (17.3)	57.0	0.13

	HaplotypeB	401 (36.9)	80 (7.4)	26.1	0.27	541 (42.1)	88 (6.8)	28.0	0.14

SNP = Single Nucleotide Polymorphism

HWE = Hardy Weinberg Equilibrium

MAF = Minor Allele Frequency

NFE2L2 = Nuclear Factor (Erythroid-derived 2)-Like 2

KEAP1 = Kelch-like ECH-associated protein-1

Five *NFE2L2 *TNR alleles, including three alleles not observed previously [22] i.e. 2, 6 and 7 CCG repeats, were identified in the Doetinchem cohort. These three novel alleles occurred with a total cumulative frequency of 0.4% (see additional file 1 for details).

**Figure 1:**
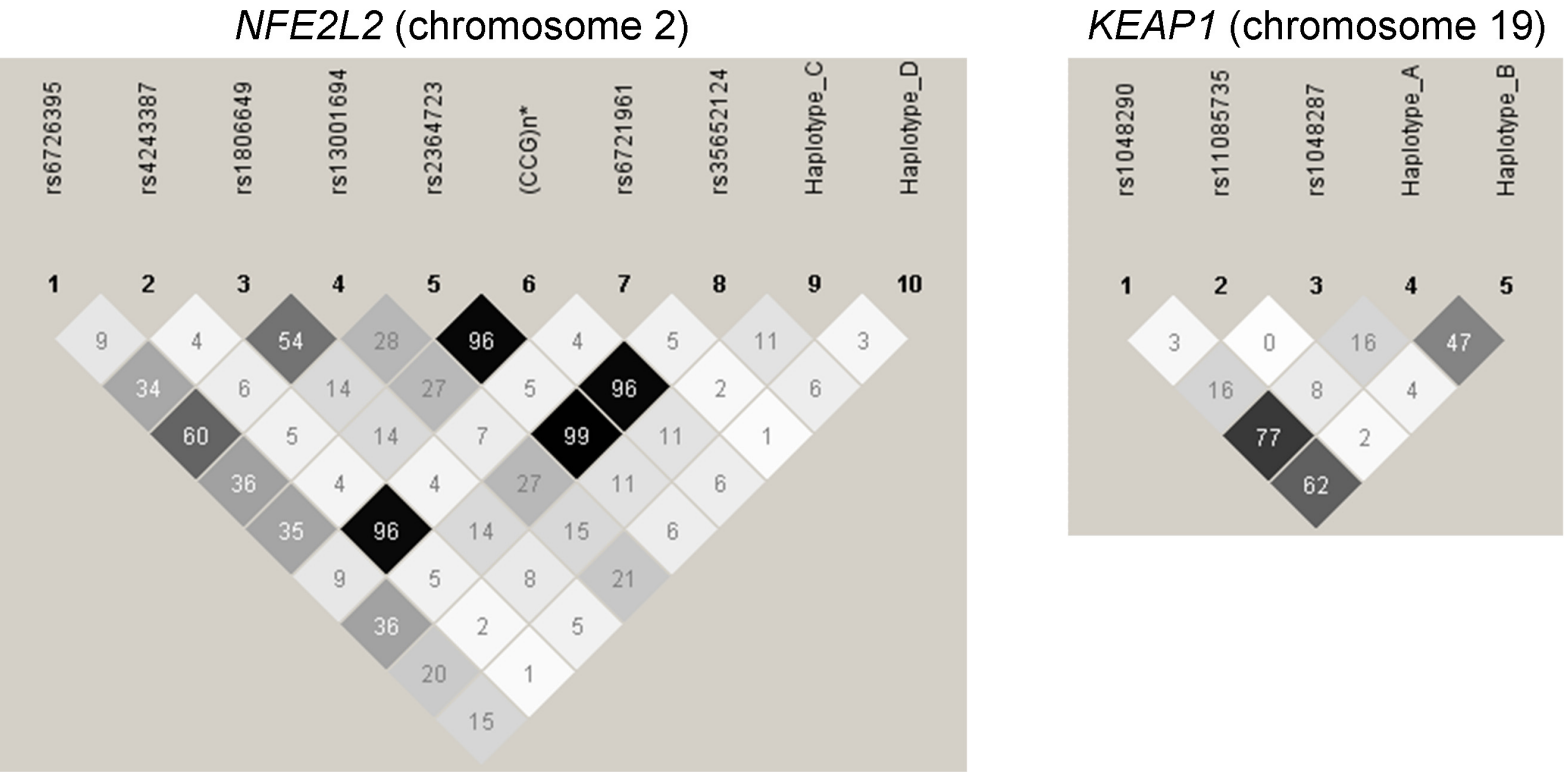
***NFE2L2 *and *KEAP1 *linkage disequilibrium plots (100·r^2^) in the Doetinchem cohort (n = 1,152)**. *given for the wild type (5 CCG repeats) and the mutant (4 CCG repeats) allele *NFE2L2 *= *Nuclear Factor Erythroid 2-Like 2 KEAP1 = Kelch-like ECH-associated protein-1*.

The *NFE2L2 *G(-686)A (rs35652124) SNP, CCG TNR and rs2364723 SNP were in high LD as well as *NFE2L2 *C(-650)A (rs6721961) and rs4243387 SNPs (r^2 ^≥ 0.96, figure 1). We observed 5 prevalent (>5% frequency) haplotypes in *NFE2L2*, and 4 prevalent haplotypes in *KEAP1 *in both cohorts (table 3). Two haplotypes in *NFE2L2 *(haplotypes C and D) were unique, i.e. they were not tagged by a single allele of any SNP (table 3). Similarly, 2 haplotypes in *KEAP1 *(haplotypes A and B) were unique (table 3).

**Table 3:** Characteristics of *NFE2L2 *and *KEAP1 *haplotypes occurring with >5% frequency in the two cohorts studied

**Gene**	**Haplotype**	SNP*	**Frequency [%]**	
		
		rs6726395-rs4243387-rs1806649- rs13001694-rs2364723	**Doetinchem cohort**	**Vlagtwedde-Vlaardingen cohort**
*NFE2L2*	A	0-0-0-0-1	31.0	32.5
	
	B	1-0-1-1-0	25.0	24.5
	
	C	0-0-0-0-0	18.9	19.1
	
	D	1-0-0-1-0	12.4	13.0
	
	E	1-1-0-0-0	9.3	7.0
	
	-	Rare pooled	3.6	3.9

		rs1048290-rs11085735-rs1048287		
	
*KEAP1*	A	0-0-0	57.7	57.0
	
	B	1-0-0	26.1	28.0
	
	C	1-0-1	9.8	9.7
	
	D	0-1-0	5.5	4.9
	
	-	Rare pooled	0.9	0.4

*0/1 corresponds to the major/minor allele of SNPs

NFE2L2 = Nuclear Factor (Erythroid-derived 2)-Like 2

*KEAP1 = Kelch-like ECH-associated protein-1*.

### *NFE2L2 *and *KEAP1 *variations and level of FEV_1_

SNP rs2364723 in *NFE2L2 *was associated (p = 0.06) with a lower FEV_1 _level, and SNP rs11085735 in *KEAP1 *was significantly associated with a higher FEV_1 _level in the Vlagtwedde-Vlaardingen cohort (table 4). Similar, but non-significant trends for an additive effect were observed in the Doetinchem cohort, resulting in significant effects in the pooled cohort analysis (table 4).

**Table 4:** Additive effects of genetic variations in *NFE2L2 *and *KEAP1 *on the level of FEV_1_

**Gene**	**Variation**	**Doetinchem cohort**			**Vlagtwedde-Vlaardingen cohort**			**Pooled cohorts**
		
		**B [ml]**	**95% CI**	**p**	**B [ml]**	**95% CI**	**p**	**p**
*NFE2L2*	rs6726395	-13.8	-51.0 – 23.4	0.47	14.1	-17.9 – 46.1	0.39	0.827
	
	rs4243387	0.2	-61.9 – 62.3	0.99	20.6	-36.5 – 77.7	0.48	0.620
	
	rs1806649	-44.5	-87.3 – -1.7	**0.04**	0.2	-36.7 – 37.1	0.99	0.150
	
	rs13001694	-20.9	-58.7 – 16.9	0.28	13.0	-19.5 – 45.5	0.43	0.948
	
	rs2364723	-22.9	-63.6 – 17.8	0.27	-32.1	-65.4 – 1.2	0.06	**0.026**
	
	Haplotype C	44.8	-3.4 – 93.0	0.07	24.3	-17.8 – 66.4	0.26	**0.040**
	
	Haplotype D	47.0	-12.1 – 106.1	0.11	21.2	-29.7 – 72.1	0.41	0.064

*KEAP1*	rs1048290	12.4	-26.3 – 51.1	0.53	-6.5	-40.8 – 27.8	0.71	0.784
	
	rs11085735	69.9	-9.3 – 149.1	0.08	97.1	22.4 – 171.8	**0.01**	**0.003**
	
	rs1048287	-11.9	-70.8 – 47.0	0.69	-33.2	-86.3 – 19.9	0.22	0.287
	
	Haplotype A*	23.9	-13.8 – 61.6	0.21	7.0	-26.5 – 40.5	0.68	0.206
	
	Haplotype B	8.9	-33.2 – 51.0	0.68	4.6	-32.5 – 41.7	0.81	0.601

Significant p values are depicted in bold

* for the recessive effect: B = 76.8 ml (95% CI: 8.0–145.6), p = 0.03 (Doetinchem cohort) and B = 45.1 ml (95% CI: -15.5–105.7) p = 0.14 (Vlagtwedde-Vlaardingen cohort); p = 0.01 in the pooled cohort analysis

Parameter estimate B (corresponding to the "per-allele" effect on the level of FEV_1 _in ml), its 95% Confidence Interval and p value are estimated for genetic variations in *NFE2L2 *and *KEAP1 *using Linear Mixed Effect model analysis on FEV_1 _level adjusted for genotypes (coded: 0 = homozygotes wild type, 1 = heterozygotes, 2 = homozygotes mutant) packyears smoked, sex, age, height and correlation of FEV_1 _measurements within subjects and cohort binary variable for the pooled cohorts analysis.

NFE2L2 = Nuclear Factor (Erythroid-derived 2)-Like 2

KEAP1 = Kelch-like ECH-associated protein-1

FEV_1 _= Forced Expiratory Volume in 1 second

CI = Confidence Interval

**Figure 2:**
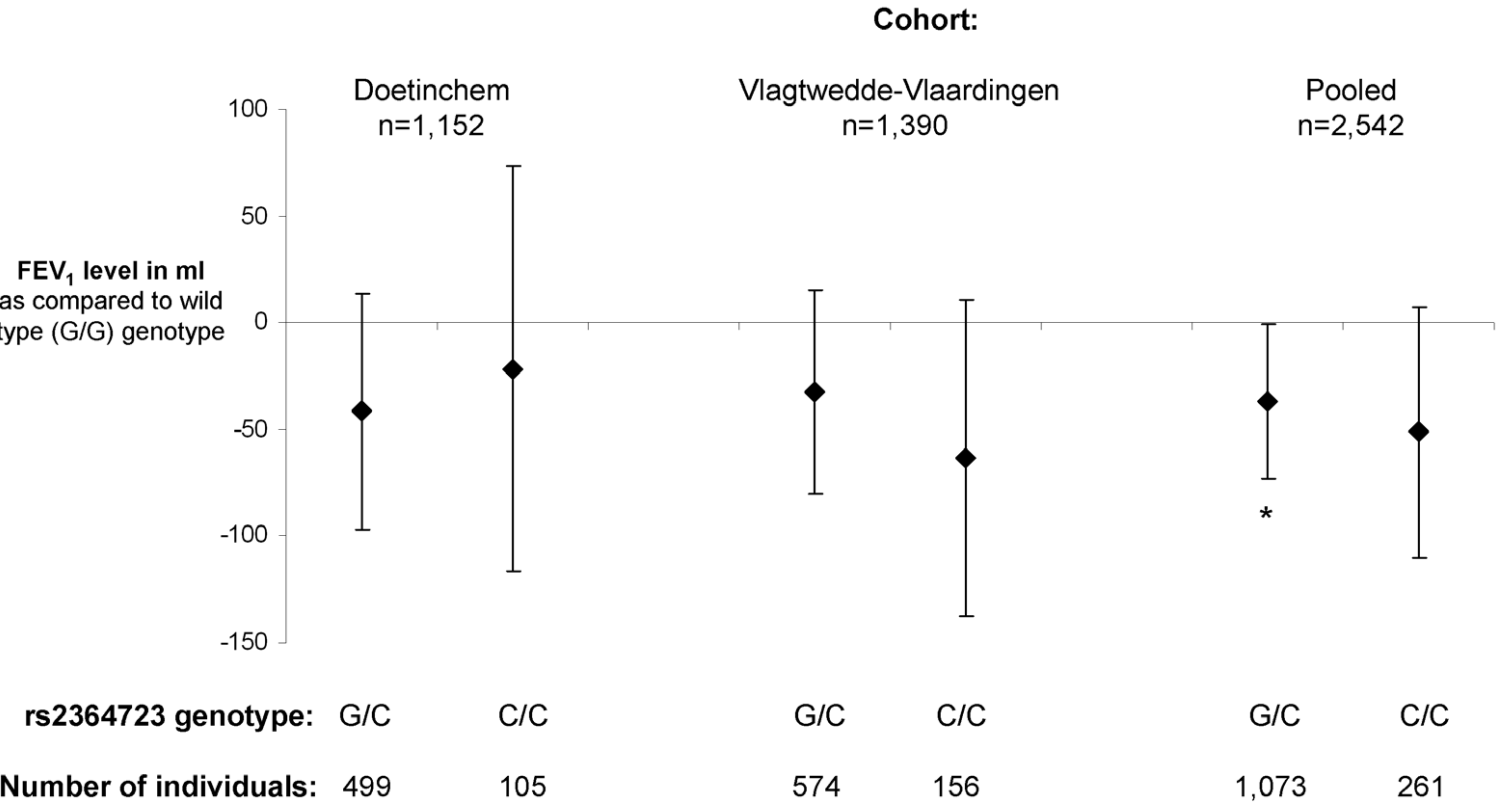
**Mean adjusted FEV_1 _level for heterozygote and homozygote mutant genotypes of the *NFE2L2 *rs2364723 SNP as compared to wild type**. Mean adjusted effects (squares) and corresponding 95% Confidence Intervals (bars) are presented. *p < 0.05 as compared to wild type. *NFE2L2 *= *Nuclear Factor Erythroid 2-Like 2*. FEV_1 _= Forced Expiratory Volume in 1 second.

**Figure 3:**
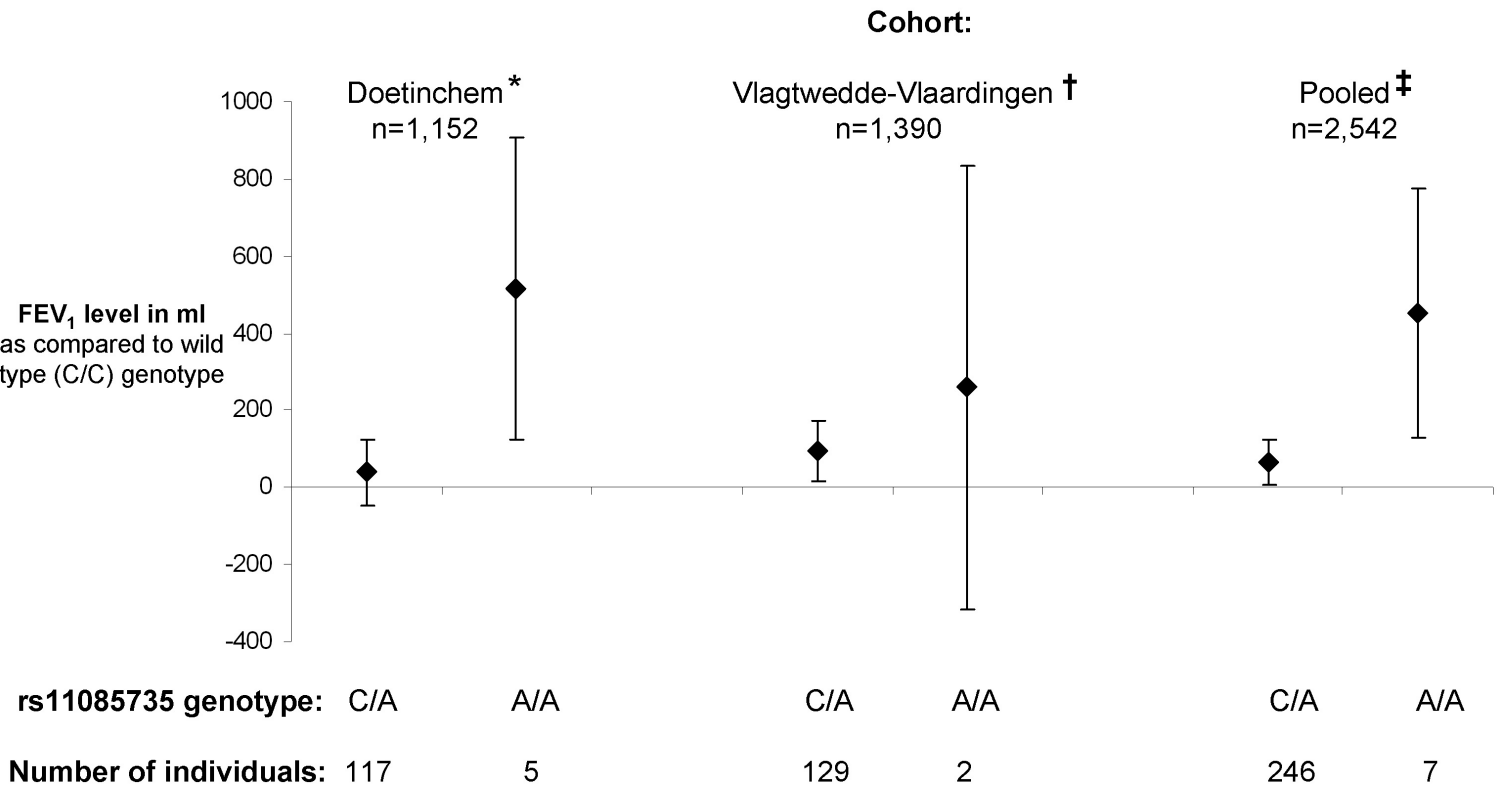
**Mean adjusted FEV_1 _level for heterozygote and homozygote mutant genotypes of the *KEAP1 *rs11085735 SNP as compared to wild type**. Mean adjusted effects (squares) and corresponding 95% Confidence Intervals (bars) are presented. * p < 0.05 for homozygote mutant genotype as compared to wild type or heterozygotes. ^† ^p < 0.05 for heterozygote genotype as compared to homozygote wild type or homozygote mutant. ^‡ ^p < 0.05 for all between-genotype comparisons. *KEAP1 = Kelch-like ECH-associated protein-1*. FEV_1 _= Forced Expiratory Volume in 1 second

Heterozygote subjects for rs2364723 SNP had a significantly lower FEV_1 _level as compared to homozygote wild type subjects (figure 2), while for the rs11085735 SNP all between-genotypes differences were significant in the pooled cohort analysis (figure 3).

Haplotype C in *NFE2L2 *was associated with higher FEV_1 _levels using an additive model in the pooled cohort analysis exclusively (table 4). Haplotype A in *KEAP1 *was associated with higher FEV_1 _level in a recessive model in the pooled cohort analysis (table 4). No additional consistent associations were observed for other SNPs or other genetic models (data not shown).

#### Interaction between SNPs in NFE2L2 and KEAP1

There was no significant interaction between SNPs in *KEAP1 *and *NFE2L2 *(using combinations of dominant and/or additive effects) in relation to the level of FEV_1 _in the pooled cohort analysis (data not shown).

### Interaction between smoking and *NFE2L2 *and *KEAP1 *variations and level of FEV_1_

We observed no significant interaction between ever/never smoking status and variations in *NFE2L2 *or *KEAP1 *in relation to the level of FEV_1_. Yet the effect of rs11085735 in *KEAP1 *was significant only in never smokers, while the effect of rs2364723 and haplotype C in *NFE2L2 *was significant only in ever smokers (table 5). In the pooled cohort analysis we observed significant interactions between packyears smoked with two linked variations in *KEAP1 *i.e. rs1048290 (B_INT _= 1.9 ml/(packyear*allele number) SE_INT _= 0.9 p = 0.03) and haplotype B (B_INT _= 1.9 ml/(packyear*allele number) SE_INT _= 0.9 p = 0.04). In the single cohort analysis these interaction terms were not significant (p > 0.10 for both cohorts).

**Table 5:** Additive effects of *NFE2L2 *and *KEAP1 *SNPs on the level of FEV_1 _in never- and ever-smokers

		**Doetinchem cohort (n = 1,152)** *second survey*						**Vlagtwedde-Vlaardingen cohort (n = 1,390)** *last survey*					
		
**Gene**	**Variation**	**Never smokers**			**Ever smokers**			**Never smokers**			**Ever smokers**		
		
		**B [ml]**	**SE**	**p**	**B [ml]**	**SE**	**p**	**B [ml]**	**SE**	**p**	**B [ml]**	**SE**	**p**
*NFE2L2*	rs6726395	-33.1	34.1	0.33	-3.4	24.2	0.89	9.7	28.3	0.73	6.4	22.7	0.78
	
	rs4243387	15.5	55.5	0.78	-1.2	41.1	0.98	3.9	56.0	0.94	12.8	38.6	0.74
	
	rs1806649	-97.1	37.7	0.01	-19.0	28.4	0.50	40.6	31.5	0.20	-13.1	26.6	0.62
	
	rs13001694	-54.5	34.7	0.12	-16.0	24.5	0.52	13.5	28.2	0.63	5.6	23.3	0.81
	
	rs2364723	21.5	37.3	0.56	-36.6	26.8	**0.17**	-3.2	28.4	0.91	-36.2	24.0	**0.13**
	
	Haplotype C	3.5	44.6	0.94	54.2	31.3	**0.08**	-22.9	35.2	0.52	47.5	30.5	**0.12**
	
	Haplotype D	86.7	59.1	0.14	19.5	37.0	0.60	-40.3	42.5	0.34	38.7	37.1	0.30

*KEAP1*	rs1048290	-0.8	35.3	0.98	17.0	25.5	0.51	-21.8	29.2	0.46	44.6	24.6	0.07
	
	rs11085735	116.2	75.8	**0.13**	64.6	50.4	0.20	112.2	60.8	**0.07**	23.5	54.5	0.67
	
	rs1048287	-25.0	53.8	0.64	-10.4	38.7	0.79	-56.8	44.0	0.20	5.1	38.6	0.90
	
	Haplotype A*	25.8	34.0	0.45	22.1	24.9	0.38	4.8	28.8	0.87	36.7	23.8	0.12
	
	Haplotype B	12.3	38.3	0.75	2.3	27.8	0.93	3.6	30.7	0.91	46.3	26.7	0.08

P values depicted in bold indicate associations significant (p < 0.05) in the pooled cohort analysis within never- or ever-smokers.

*p < 0.05 for a positive recessive effect in ever and never smokers in the pooled cohort analysis (p > 0.05 for the analysis concerning separate cohorts)

None of the SNP in any model showed significantly different effect between never and ever smokers as tested with the pooled cohort linear regression analysis containing interaction term (binary variable reflecting smoking status) adjusted for height, sex, age, cohort and ever/never smoking status. Two underlined *KEAP1 *variations showed a significant interaction with packyears smoked in an additive model within ever-smokers in the pooled cohort analysis:

rs1048290: B_INT _= 1.9 ml/(packyear*number of alleles) SE_INT _= 0.9 p = 0.03

Haplotype B: B_INT _= 1.9 ml/(packyear*number of alleles) SE_INT _= 0.9 p = 0.04

### SNPs in *NFE2L2 *and *KEAP1 *and course of FEV_1_

We did not observe any significant effect of SNPs in *NFE2L2 *and/or *KEAP1 *on the course of FEV_1 _in either of the cohorts nor in the pooled cohort analysis for any genetic model tested (see table 6 for additive effects).

**Table 6:** Additive effects of genetic variations in *NFE2L2 *and *KEAP1 *on the longitudinal course of FEV_1_

**Gene**	**Variation**	**Doetinchem cohort**			**Vlagtwedde-Vlaardingen cohort**			**Pooled cohorts**
		
		**B [ml/yr]**	**95% CI**	**p**	**B [ml/yr]**	**95% CI**	**p**	**p**
*NFE2L2*	rs6726395	0.2	-2.5 – 2.9	0.88	0.1	-1.5 – 1.7	0.90	0.873
	
	rs4243387	-1.2	-5.6 – 3.2	0.60	-1.9	-4.8 – 1.1	0.21	0.106
	
	rs1806649	1.5	-1.6 – 4.5	0.35	1.0	-1.0 – 3.0	0.31	0.151
	
	rs13001694	0.0	-2.7 – 2.7	1.00	0.7	-1.0 – 2.4	0.40	0.337
	
	rs2364723	-0.3	-3.2 – 2.6	0.84	-0.6	-2.3 – 1.1	0.50	0.368
	
	Haplotype C	-0.3	-3.8 – 3.1	0.85	0.9	-1.3 – 3.1	0.40	0.401
	
	Haplotype D	-2.3	-6.6 – 2.0	0.29	0.0	-2.6 – 2.5	0.98	0.627

*KEAP1*	rs1048290	-2.0	-4.7 – 0.8	0.16	1.0	-0.8 – 2.8	0.28	0.907
	
	rs11085735	3.6	-2.1 – 9.4	0.22	-0.7	-4.7 – 3.3	0.72	0.774
	
	rs1048287	-1.8	-5.9 – 2.4	0.41	-0.4	-3.1 – 2.4	0.80	0.614
	
	Haplotype A	-1.3	-4.0 – 1.4	0.35	0.8	-1.0 – 2.5	0.38	0.817
	
	Haplotype B	-1.6	-4.6 – 1.4	0.30	1.5	-0.5 – 3.4	0.14	0.573

Parameter estimate B (corresponding to the "per-allele" effect on the change in FEV_1 _in ml/yr), its 95% Confidence Interval and p value are estimated for genetic variations in *NFE2L2 *and *KEAP1 *using Linear Mixed Effect model analysis on FEV_1 _level adjusted for genotypes (coded: 0 = homozygotes wild type, 1 = heterozygotes, 2 = homozygotes mutant), age at entry, sex, packyears smoked, FEV_1 _level at baseline (and their interaction with time) and correlation of lung function measurements within subjects (random factor assigned to the intercept and time) and cohort binary variable for the pooled cohorts analysis.

NFE2L2 = Nuclear Factor (Erythroid-derived 2)-Like 2

KEAP1 = Kelch-like ECH-associated protein-1

FEV_1 _= Forced Expiratory Volume in 1 second

CI = Confidence Interval

## Discussion

The current study shows that polymorphisms in antioxidant transcription factor *NFE2L2 *and its repressor *KEAP1 *affect the level of FEV_1 _in the general population.

NFE2L2 is required for the transcription initiation of many antioxidant-related genes including candidate genes for lung excess function loss and COPD development such as *Heme Oxygenase 1 *and *Glutamate Cysteine Ligase *[11,27,36]. Moreover, murine models have shown that the *Nfe2l2 *depletion *in vivo *results in elastase- [17] and cigarette smoke-induced [18] emphysema development. Thus a functional genetic impairment concerning *NFE2L2 *and/or its cytosolic repressor *KEAP1 *would likely result in detrimental consequences *in vivo*.

It has been shown that lung function is genetically determined [2,3], however so far only low-prevalent polymorphisms have been consistently associated with COPD development across independent studies, i.e. the Glu342Lys substitution in *SERPINA1 *(frequency 1%–3% in Caucasians) that leads to a1-antitrypsin deficiency [6-8] and the Arg213Gly substitution in *Superoxide Dismutase 3 *(frequency 1%–2% in Caucasians) [9,10], suggesting that low-prevalent SNPs are important contributors to COPD development. Detection of the effect provided by such low prevalent SNPs often requires large sample sizes, even when the effect size is substantial. Similarly, small genetic effects for highly prevalent variations, such as those genotyped in the current study, need to be assessed in large sample sizes. Therefore, we used all available FEV_1 _measurements in both cohorts, in order to achieve the highest possible statistical power. Moreover, we additionally performed analyses on the pooled cohorts including over 2,500 subjects with over 11,000 FEV_1 _measurements.

In our opinion the most convincing association shown in the current study was that the rs11085735 SNP in *KEAP1 *significantly associated with higher FEV_1 _levels in the pooled cohort as well as in both cohorts analyzed separately, yet using different genetic models. This SNP is located in the intron 3 of *KEAP1*, relatively close (73 bp) to the exon 3 of this gene, and thus it might have functional consequences e.g. via affecting *KEAP1 *mRNA splicing. Haplotype A in *KEAP1 *was associated with higher FEV_1 _level in the Doetinchem cohort and in the pooled cohort analysis using a recessive model only. Since this haplotype does not tag any SNP that was investigated in the current study, it may be in linkage disequilibrium with another functional SNP that is either not known yet or is located outside the region that was selected for tagging.

SNP rs2364723 and haplotype C in *NFE2L2 *were associated with the level of FEV_1 _in the pooled cohort analysis, as caused by a similar though not significant trends present in both cohorts. SNP rs2364723 is in almost complete LD with the recently described promoter polymorphisms i.e. G(-686)A (rs35652124) and CCG Trinucleotide repeat (figure 1) [22], implicating a role in the regulation of *NFE2L2 *transcription. We found no evidence for an association of another previously identified functional *NFE2L2 *SNP (i.e. C(-650)A (rs6721961) tagged by us with rs4243387 SNP) [23].

None of the analyzed genetic variations showed a significantly different effect on the level of FEV_1 _between never and ever smokers, yet the effects provided by *NFE2L2 *rs2364723 SNP and haplotype C were more prominent in ever smokers while the effect of *KEAP1 *rs11085735 SNP was significant in never smokers exclusively. Interestingly another variation in *KEAP1 *(i.e. rs1048290 linked with haplotype B) showed a protective effect on the level on FEV_1 _in interaction with packyears smoked within ever smokers. The observed association of the level of FEV_1 _and the interaction between rs1048290 SNP and smoking can be somewhat weakened by a deviation from HWE observed for this SNP in one of the cohorts studied. Since the common cause of such deviation is a genotyping error, we have genotyped another, completely correlated, rs9676881 SNP, which also showed significant deviation from HWE. This suggests that genotyping error was not a cause of the observed deviation from HWE. Significant results obtained in the analysis stratified by smoking status (ever and never smokers), or in the gene by packyears interaction analysis did not reach significance in either of the cohorts analyzed separately. Since this could be due to insufficient power provided by single cohorts, subsequent studies are warranted.

Using publicly available data on the British 1958 Birth cohort [35], we checked whether our results on the significant association of SNPs with the level of FEV_1 _could be replicated in this independent population. The additive effects provided by. rs11085735 in *KEAP1 *and rs2364723 in *NFE2L2 *were not significant, p values being 0.11 and 0.59–0.70 (depending on the genotyping method) respectively. However, both associations were in the same direction as found in our two Dutch cohorts, i.e. positive for rs11085735 in *KEAP1 *(B = 52.7 ml/allele, 95% Confidence Interval (CI) = -12.6 – 118.0) and negative for rs2364723 in *NFE2L2 *(B = -7.3 ml/allele, 95% CI = -44.3 – 29.6, representing higher p value). A subsequent meta-analysis of the Doetinchem, Vlagtwedde-Vlaardingen and British 1958 Birth cohorts showed a higher significant protective effect of the *KEAP1 *SNP on the level of FEV_1 _(p = 0.0008) as compared to the pooled analysis in the two Dutch cohorts (p = 0.003, table 4). The p value of the additive and detrimental effect of the rs2364723 SNP was significant as well (0.036–0.046, depending on the genotyping technology in the British 1958 Birth Cohort), yet higher than the p value provided by the pooled analysis in the two Dutch cohorts (i.e. p = 0.026, table 4).

## Conclusion

Our study performed in two independent Dutch cohorts shows that genetic variations in *KEAP1 *and *NFE2L2 *affect the level, but not the longitudinal course of FEV_1 _in the general population. Therefore, it remains for future considerations whether these SNPs play a role in the development or growth of the lung. Given the importance of both genes in the regulation of oxidative stress in the lung, further studies focusing on the *NFE2L2-KEAP1 *pathway are warranted.

## Competing interests

MS has no conflict of interest to disclose. DSP has no conflict of interest to disclose. JMAB has no conflict of interest to disclose. GvdS has no conflict of interest to disclose. JPS has no conflict of interest to disclose. HAS has no conflict of interest to disclose. HMB has no conflict of interest to disclose.

## Authors' contributions

MS wrote the manuscript. MS, JPS, and HMB analyzed the data. HAS and JMAB designed the Doetinchem cohort study and managed the data. JPS designed the Vlagtwedde-Vlaardingen cohort study and managed the data. GvdS participated in the genotyping process. MS, JPS, MB, JMAB, HAS, and DS interpreted the data. All authors proposed corrections and approved the final version of the manuscript.

## Supplementary Material

Additional file 1 **SNP-selection and NFE2L2 genotyping** Click here for file
