# Supplementary material for: Level and course of FEV1 in relation to polymorphisms in NFE2L2 and KEAP1 in the general population
Source: Respir Res. 2009 Aug 11;10(1):73. doi: 10.1186/1465-9921-10-73 (PMC2738671; doi:10.1186/1465-9921-10-73)
Supplement: Additional file 1 — SNP-selection and NFE2L2 genotyping [file 1465-9921-10-73-S1.pdf]

Additional file 1

Genotyping

The three *NFE2L2* novel polymorphisms were genotyped in the Doetinchem cohort only since they were highly correlated (r^2>0.96) with certain SNPs derived from HapMap, and consequently did not provide different information than the HapMap-derived SNPs. Since rs1048290 SNP in *KEAP1* showed a significant deviation from Hardy Weinberg Equilibrium in Vlagtwedde-Vlaardingen cohort we additionally genotyped rs9676881 SNP, which is in complete linkage disequilibrium with rs1048290 SNP (r^2=1.0 according to HapMap CEU genotype data (release 23a/phase II, March 2008)). Since both SNPs are located relatively far from each other (3.7kb) this allowed us to check whether the observed HWE deviation could occur due to genotyping errors.

Amplification of the region spanning Trinucleotide CCG Repeat (TNR) in the *NFE2L2* was performed by 10 µl of PCR reaction containing:

- 10 ng of the DNA
- 2 µl DMSO
- 1 µl $MgCl_2$ (25 mM)
- 1 µl dNTPs (2mM)
- 0.1 µl AmpliTaq® (Applied Biosystems, USA) DNA polymerase and 1 µl AmpliTaq® buffer
- 0.5 µl of forward (5'-FAM-atgagctgtggaccgtgtgt) and reverse (5'-gcgtgtagccgattaccgagt) primers (10 µM each)
- water to the final volume of 10 µl

PCR amplification consisted of the following steps:

- o Initial denaturation for 5 min. at 95.0^{0}C
- o 35 cycles according to the protocol:
  - o Denaturation for 45 sec. at 94.0^{0}C
  - o Annealing for 30 sec. at 57.9^{0}C
  - o Elongation for 30 sec. at 72.0^{0}C
- o Final elongation for 5 min. at 72.0^{0}C

The PCR product comprising the TNR was labeled with a fluorescent FAM dye, present in the forward primer. It basically had two possible lengths (i.e. 156 and 159 base pair) corresponding to the two most frequent TNR alleles (i.e. 4 and 5 CCG repeats) present in our populations. The PCR reaction was followed by MegaBACE electrophoresis of the product in an acryl amide gel in the presence of sizer. MegaBACE Genetic Profiler software (version 2.2, GE Healthcare, UK) was used to assign the specific TNR alleles to the fluorescent peaks (figure 1). To check for the quality of genotyping we repeated the genotyping process in 100 samples taken from stock vials, with no genotyping errors detected.

SNP x time interaction analysis with Linear Mixed Effect models

The introduction of SNP x time (defined in relation to the first FEV_1 measurement and with a random effect assigned) interaction term to the model and additional adjustment for age at entry and FEV_1 level at entry (and exclusion of height) allowed us to test how SNPs associate with FEV_1 course over time. In this analysis FEV_1 measurements (n=6,966) were included from the age of 30 years in the Vlagtwedde-Vlaardingen cohort, because an individual's maximal achieved lung function is

assumed to have been reached before that age and the level of lung function is considered to be either in the plateau or decline phase [1]. In the Doetinchem cohort all subjects crossed the 30 years of age at the second survey and were thus included to the analysis.

FIGURES

Figure 1: Genetic profiler output plots for all *NFE2L2* TNR genotypes found in the Doetinchem cohort (n=1,152).

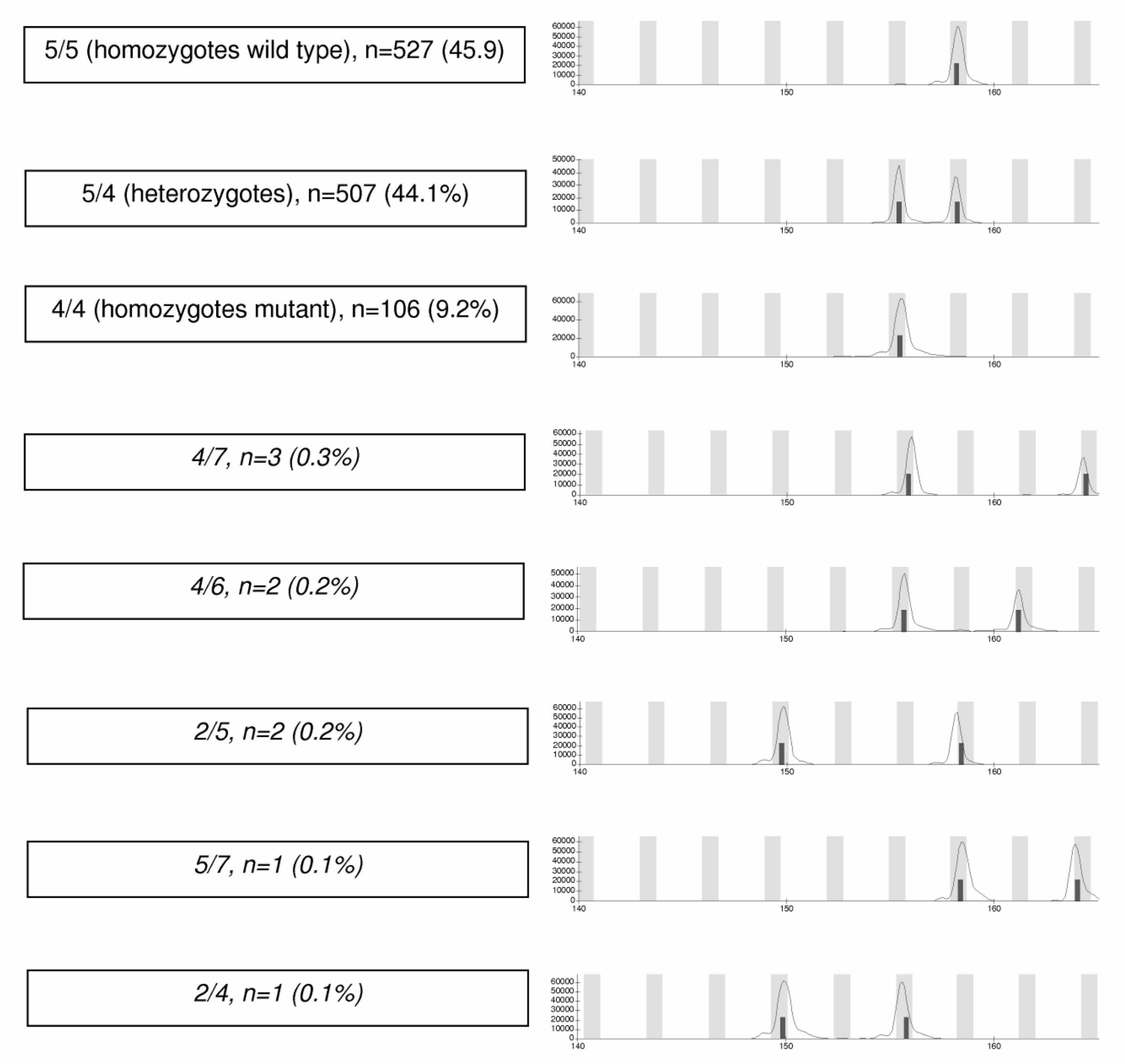

Numbers of individuals and frequencies are given for all common and rare (italics) genotypes represented by number of TNR alleles.

NFE2L2 = Nuclear Factor (Erythroid-derived 2)-Like 2

TNR=Trinucleotide CCG Repeat in *NFE2L2*

References

1. Rijcken B, Weiss ST: **Longitudinal analyses of airway responsiveness and pulmonary function decline.** *Am J Respir Crit Care Med* 1996, **154:**S246-S249.
